# Electrochemical Detection of Microplastics in Aqueous Media

**DOI:** 10.3390/s25144278

**Published:** 2025-07-09

**Authors:** Mashrur Sakib Choyon, Sindre Søpstad, Martin Peacock, Hamed Salmani, Erik Johannessen

**Affiliations:** 1 Department of Microsystems, University of Southeastern Norway, 3184 Borre, Norway; mashrurchoyon@gmail.com (M.S.C.); hamed.salmani@usn.no (H.S.); 2 Zimmer & Peacock, 3125 Tonsberg, Norway; sindre@zimmerpeacock.com (S.S.); martin.peacock@zimmerpeacock.com (M.P.)

**Keywords:** microplastics, electrochemistry, sensor, particle count, impact, frequency domain

## Abstract

Microplastics in aqueous media can be detected through transient oxygen reduction from impacts with an electrified carbon-coated microwire. Each impact is recorded as a spike count in the time domain or as prominent peaks in the frequency domain. The spike count increased from approx. 60 s^−1^ (pure solution) to 90 s^−1^ (with microplastics) and 230 s^−1^ (microplastics in deoxygenated solutions), whereas the frequency domain revealed the presence of spikes in the 7, 21, and 24 Hz regions. The spike count showed a co-variance with the concentration of microparticles, with a linear detection range from 0.02% (*w/v*) to 0.04% (*w/v*). The electrochemical sensor, characterized by its simple and cost-effective design, may provide a rapid and user-friendly method for the detection of microplastics.

## 1. Introduction

Synthetic polymer materials such as plastics and their composites are the main ingredients in most industrial as well as consumer products being manufactured globally. Introduced as “Bakelite” in 1907, which was synthesized from a mixture of phenol and formaldehyde, this new class of materials was found to revolutionize the industry with their combined strength, light weight, corrosion resistance, and low cost [1,2]. Today, plastics are synthesized mainly from oil and gas, reaching production numbers as high as 390.7 million tons annually [3]. Such a quantity, combined with inadequate waste disposal routines, has created a significant pollution challenge affecting the global environment [4,5].

Plastics decompose by hydrolysis, as well as by photo-, thermal-, and thermo-oxidative degradation [6]. The photo-oxidative degradation of polymers, which frequently occurs on beaches or shorelines, is initiated by UV-B light and involves chemical processes such as chain scission and chain transfer, leading to the breakdown of polymer chains into smaller fragments [6]. The results are particles less than 5 mm in length called “microplastics” or “nanoplastics”, which may accumulate in soil, water, and the atmosphere for centuries before they are completely broken down into their base constituents [7]. Thus, health concerns are raised by the evidence of microplastics found in drinking water, with concentrations ranging from 1 × 10^−2^ to over a million pieces/m^3^ [8,9]. When ingested and built up in the food chain, microplastics may pose a significant threat to human health as well as terrestrial and marine ecosystems [2,10,11] ranging from zooplankton to whales [12]. Further, the detection of microplastics in rainfall suggests that they have also become airborne, which may explain their presence in honey and beverages [13,14].

The effect of microplastics on living species is not known, but studies suggest that they may have an impact on gene expression, intestinal damage, and reproduction [15,16]. The impact is influenced by various parameters, such as the chemical composition, size, structure, and kind of polymer. For example, while polyethylene and polypropylene are less reactive, chemical additions in polymers like polyurethane and PVC have the potential to cause cancer or mutagenicity [17,18]. Because they can enter cells and tissues, smaller microplastics, especially nanoplastics, may pose a greater risk [19,20]. These have been shown to have the ability to penetrate intestinal membranes and accumulate in tissues, such as the human placenta [21], where they may lead to immunological reactions, oxidative stress, neurological impairments, reproductive issues, and transgenerational toxicity [16].

A study found that the microplastic ingestion rates in *Neocalanus cristatus* and *Euphausia pacifica* correlated with their dietary preferences and proximity to shore [22]. This trophic transfer allows microplastics to ascend the marine food chain. Microplastics were detected in 36.5% of the fish examined, with rayon and polyamide being the most prevalent types [23].

Similar to particulate air pollution, microplastics can induce oxidative stress, inflammation, and other adverse health effects [24]. Microplastics have the ability to spread throughout different tissues and enter the lymphatic and circulatory systems. There are serious health concerns regarding their potential to carry and release hazardous chemicals [25,26,27].

Most marine litter comes from land-based sources which are distributed through freshwater systems, and account for 80% of the plastic waste found in the oceans [6,28,29,30]. The origins of this plastic waste are listed in Table 1.

There are no current universal methods for identifying microplastics in water [9]. Most of the conventional detection is performed by Fourier Transform Infrared (FTIR) spectroscopy [31], which depends on the selective absorption of covalent bonds towards particular wavelengths. This results in changes in vibrational energy that are seen as transmittance patterns in the FTIR spectrum [32]. Another conventional method is Raman spectroscopy, which examines vibrational energy modes by using scattered light [33,34]. An overview of the various methods for detecting microplastics and their distinguishing features is presented in Table 2.

Beyond the conventional techniques, portable devices based on a CCD camera have been made to detect PET and LDPE polymers based on the evaluation of their curved shapes [35]. Combined with Raman spectroscopy, concentrations as low as 0.015% (*w/v*) have been detected [36]. In another study, polymer composition was analyzed through heat stability or degradation studies [37], and surface plasmon resonance (SPR) was used as a stand-alone detection system, primed with estrogen-sensitive capturing probes to identify particles as small as 20 µm. A chromatographic analysis revealed that estrogen receptors (ERs) on the SPR sensor influenced microplastic elution times based on the surface charge. Distinct patterns on the SPR sensorgram indicated microplastic interactions with ERs, and the linear relationship between the bound microplastics and SPR response units enabled an accurate quantification [38].

**Table 2:** Traditional techniques for detecting and analyzing microplastics [9].

Method	Technique	Methodology	Particle Size	Advantages	Disadvantages
Visual	Microscopic counting [39]	Particle count, hemocytometer	~µm	Quick ID if large MP cons	Composition unknown
Spectroscopic	FTIR [40]	IR radiation, molecular struct	20–500 µm	Fast, reliable, non-destructive	Requires IR-reactive samples
	Raman spectroscopy [41]	Laser-induced Raman shift	1–20 µm	Min. water interference, fast	Fluorescence, impurities error
	SEM [42]	Surface images electron beam	~µm	High-resolution images	Conductive coating
Chromatographic	Thermal (Pyrolysis) [43]	Heat releases gases by GC-MS	>500 µm	Analyzes plastics/additives	Single particle per run
	HPLC [42]	Molar mass via chromatography	>mg	High recovery rates	Limited to specific polymers
Other Methods	Tagging method [44]	Fluorescence	~µm	Cost-effective screening	Error from other particles

Given this global threat of pollution, tracking plastics in terms of their micro- and nanoparticle constituents will be paramount, and one needs to develop portable solutions that can be used on site. Developing low-cost solutions would enable adaptation in the developing world, where plastic waste is a particular problem. Hence, the goal of this research is to develop a cost-effective (portable) electrochemical sensor system that can detect microplastics in water. This research expands upon the “impact concept” proposed by Shimizu et al. [45], which detects microplastics through current spikes generated by particle collisions with the electrode. However, this study introduces novel signal processing techniques, including the Fast Fourier Transform (FFT) of the Fast Amperometry (FAM) response, as an additional verification of impact events using larger sensor electrodes.

## 2. Methodology

### 2.1. Materials

The following materials were sourced from Sigma-Aldrich (St. Louis, MO, USA): NaCl (S3014), KCl (P5405), Na_2_SO_3_ (71988), and micro particles (10 µm) based on polystyrene (72986). The carbon paste (C2180626D6) was obtained from Sun Chemical (South Normanton, UK). The silver (841-7226) and copper wires (779-0729) were supplied by RS Components (Oslo, Norway). The screen-printed electrodes (ZP-HVC-SPEs) were obtained from Zimmer and Peacock (Tonsberg, Norway). The PET particles (Melinex 339) were obtained from Polymershapes (Charlotte, NC, USA) and subsequently ground down to approx. 1 mm in the laboratory. The 10 µm particles were delivered dispersed in an aqueous suspension containing trace amounts of surfactant to prevent particle agglomeration. No additional surfactants were added during the experiments.

### 2.2. Aqueous Test Solution

The test solution was made according to Shimizu et al. [45], in which 1.491 gm of KCl were dissolved in 1 L of DI water to yield a 20 mM solution, which was then kept at room temperature. The 10 µm large microplastic particles were added in the corresponding volume fractions of 0.01%, 0.02%, 0.03%, and 0.04% (*w/v*). To ensure a uniform suspension of microplastics in the solution, a magnetic stirrer was initially used for dispersion, while an ultrasonic bath was later employed to achieve better homogeneity.

### 2.3. Carbon Working Electrodes

The core principle of detection is that microplastic particles act as oxygen carriers, and when a particle collides with the electrode, the oxygen is reduced, generating a measurable signal according to the oxygen reduction reaction, ORR (Equation (1)):

(1) $$O_{2}+2H_{2}O+4e^{-}\to 4OH^{-}$$

When the working electrode (WE) is biased at the reduction potential of oxygen, the presence of dissolved oxygen produces a current response that scales with the electrode area. However, oxygen reduction signals arising from impacts with oxygen-carrying microplastics will manifest as comparably tiny variations superimposed on this signal (tens of picoamps to nanoamps). Resolving these events requires fine resolution, whereas accommodating for the dissolved oxygen (or any other signal) requires a high dynamic range. Resolution and dynamic range are opposing parameters in electronic design, resulting in a trade-off between the two. Scaling down the sensor’s working area will sufficiently shrink the unwanted background signals to a size where a low dynamic range is acceptable, and resolution is sufficient. Consequently, a commercial SPCE (ZP-HVC-SPE sensor) was modified by reducing the geometrical area of the WE from 3.8 mm^2^ to 1 mm^2^ [46]. This was supplemented with individual microwire carbon-coated electrodes with diameters down to 40 µm and a surface area down to 0.125 mm^2^.

**Figure 1:**
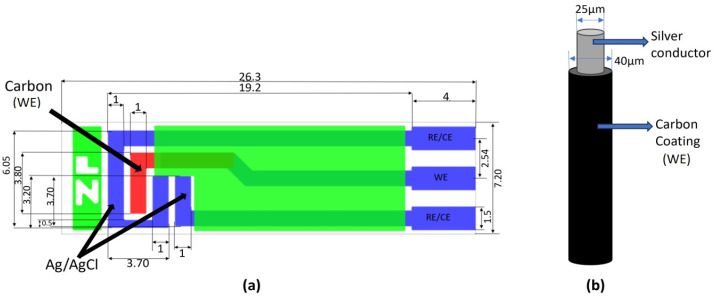
(**a**) Schematic design with dimensions of ZP-HVC-SPE (in mm) [46], (**b**) microwire carbon-coated WE with conductive metal core.

In contrast to the microwire electrode from carbon fiber (7 µm diameter, 1 mm length) utilized by Shimizu et al. [45], the present study explored the feasibility of employing readily available and cost-effective materials (copper or silver wire—commonly used in eye physiology—coated with carbon paste), mitigating the need for expensive carbon microfiber (see Table 3). Figure 1 shows the schematic diagrams of (a) the ZP-HVC-SPE and (b) the microwire carbon WE used in this project.

### 2.4. Cyclic Voltammetry Analysis

**Figure 2:**
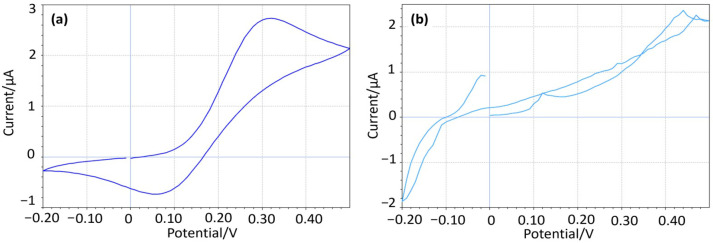
(**a**) CV showing a perfect “duck-shaped” profile, (**b**) CV from a compromised coating.

The integrity of the carbon coating was evaluated through cyclic voltammetry (CV) in a hexacyanoferrate solution characterized by the “duck-shaped” redox voltammogram. Any significant deviations, such as line intersects, suggested interference from the underlying metal substrate. Electrodes that failed to exhibit this expected “duck-shaped” response were discarded. Figure 2a shows the resultant cyclic voltammetry on an electrode with an intact carbon coating, while Figure 2b shows an equivalent curve from an electrode where the carbon coating has been compromised, permitting the exposed copper core to form oxide (Cu_2_O and CuO).

The initial CV scans were conducted against the integrated Ag/AgCl/KCl (20 mM) reference electrode of the ZP-HPVC-SPE within a potential window of −0.2 V to 0.5 V to cover the redox peaks of the hexacyanoferrate.

### 2.5. Experimental Workflow

The microwire electrodes were fabricated by dip-coating the metal wire in carbon paste, which was baked in an oven at 100 °C for 40 min, yielding a 10–20 µm thick layer. The wire was then cooled to room temperature (RT) and validated using cyclic voltammetry (Section 2.4). The wire electrode was employed as the WE and used in combination with the ZP-HVC-SPE’s counter (CE) and Ag/AgCl pseudo-reference electrodes (REs). The WE was partially submerged (approx. 1 mm) in the 10 mL test solution (with the CE and RE fully immersed). The modified WE of the ZP-HVC-SPE (Section 2.3) was used for comparison. The microwire working electrodes were cleaned with DI water and dried with compressed air for reuse after each experiment. The ORR was run at −0.4 V to reduce the offset current magnitude seen at lower potentials.

Energy-dispersive X-ray spectroscopy (EDX), combined with scanning electron microscopy (SEM), was utilized to validate the different materials employed in the electrode fabrication. Both the material composition and the relative weight percentages (wt%s) of particular components were verified using an EDX analysis.

### 2.6. Impact Signal and Background Noise

Microplastic particles made from PET (1 mm in diameter) were applied for the feasibility study to permit real-time visual observation during the tests. Deoxygenated solutions made from Na_2_SO_3_ (0.1 mg/mL) were used as controls to remove the background signal from the dissolved oxygen. A statistical spike count and FFT analysis were performed in both the oxygenated and deoxygenated solutions.

### 2.7. Signal Processing and Data Analysis

The data analysis was performed using OriginPro 2023 [47] and the Julia programming language (Version 1.8.5) [48], employing statistical and Fast Fourier Transform (FFT) techniques. The Djuli library [49] was used for the data visualization and comparison.

### 2.8. Particle Counting

**Figure 3:**
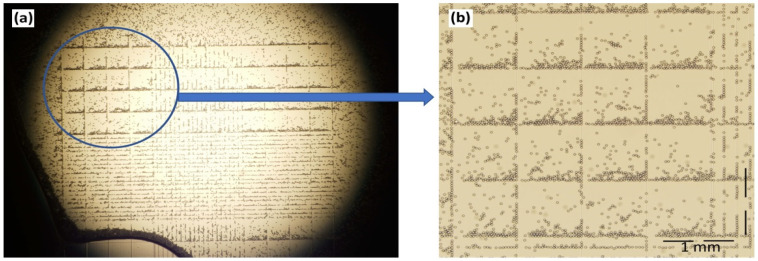
(**a**) Microplastic particles in a hemocytometer as seen under an optical microscope. (**b**) A close-up view of 16 blocks representing the statistical basis for counting.

The concentration of microplastic particles is given in terms of the weight per volume, and a translation of this into absolute numbers was made using a hemocytometer (Figure 3). It works by diluting a 100 µL solution tenfold prior to counting. The particles were counted in blocks of four and then multiplied by four. The estimated particle count per volume increased proportionally with the microplastic concentration, and a 0.01% (*w/v*) solution corresponded to 1.64 × 10^6^ particles per mL.

## 3. Results

### 3.1. The WE Dimension

The ZP-HVC-SPE electrode exhibited a baseline current of 10 nA with a peak to peak noise of ±2.5 nA, rendering it difficult to directly observe the signals induced by the microplastic impacts. By reducing the electrode area, the baseline current was reduced to 2 nA with a peak to peak noise of ±0.25 nA, from which the current spikes due to the impact events could be extracted from the FAM signals and the FFT analysis of the frequency domain.

### 3.2. Comparative Analysis of Working Electrodes

**Figure 4:**
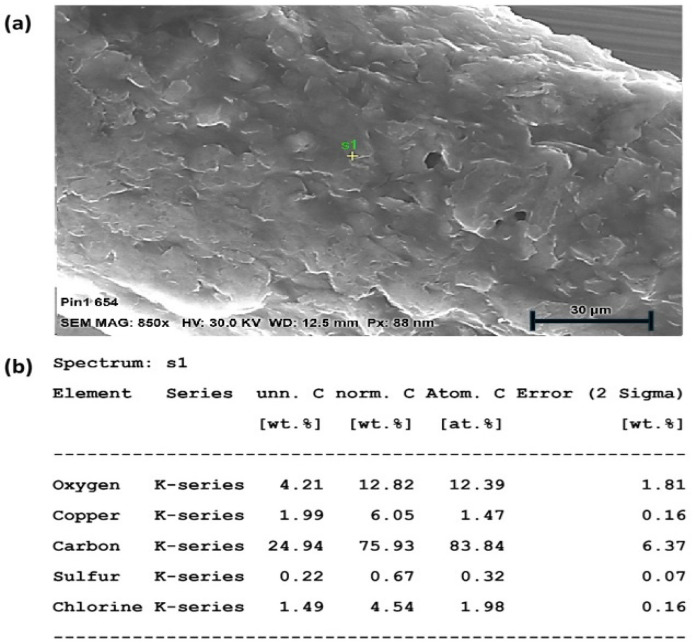
(**a**) SEM-EDX analysis of WE composition with surface micrograph of carbon coating, (**b**) material data analysis.

An SEM-EDX analysis was performed on all electrodes to confirm their material composition. The surface structure of a microwire electrode with carbon coating around a central copper conductor is shown in Figure 4. The EDX spectrum shows the presence of oxygen and carbon, which are the primary components of the carbon coating, with sulphur and chlorine as the residual components from the carbon paste and/or its solvent. The copper core is also visible, though less prominent than the carbon and oxygen, as it is buried deeper. The dimensions and material composition of the different microwire electrodes are shown in Table 3.

The EDX spectrum was acquired from a single-point analysis. This approach was used to identify the elemental composition at a representative location; while full elemental mapping was not performed in this study, the single-point analysis served as a preliminary assessment to verify the elemental constituents relevant to the sample’s surface chemistry. Imperfections in the surface coating were instead investigated in the CV analysis (Section 2.4).

### 3.3. Impact Detection Verification

**Figure 5:**
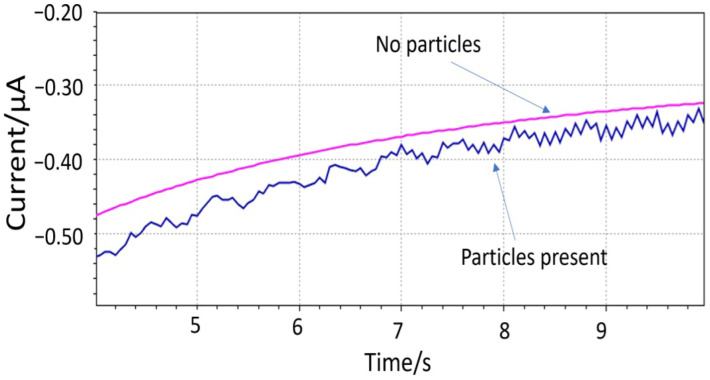
Impact events detected by microfabricated electrodes using 1 mm PET particles.

The hypothesis of the impact events was shown with the 150 µm diameter electrode (sample 2) immersed in a solution with 1 mm large PET particles (Figure 5). The PET particle concentration was estimated between 5.7 × 10^5^ and 2.8 × 10^7^ particles L^−1^. The presence of “spikes” was apparent from the noisier signal compared to the pure solution. The periodicity of the impact events is believed to be affected by the magnetic mixer. An ultrasound bath was later used for dispersing the 10 µm particles prior to the measurement. The Fast Amperometry analysis was carried out with a scan rate of 0.0005 s. The WE was biased at the lower cathodic end of where oxygen can be reduced (−0.4V vs. integrated Ag/AgCl/KCl (20 mM)) to minimize the contribution of the dissolved oxygen to the signal.

### 3.4. Statistical Analysis of Spike Count

**Figure 6:**
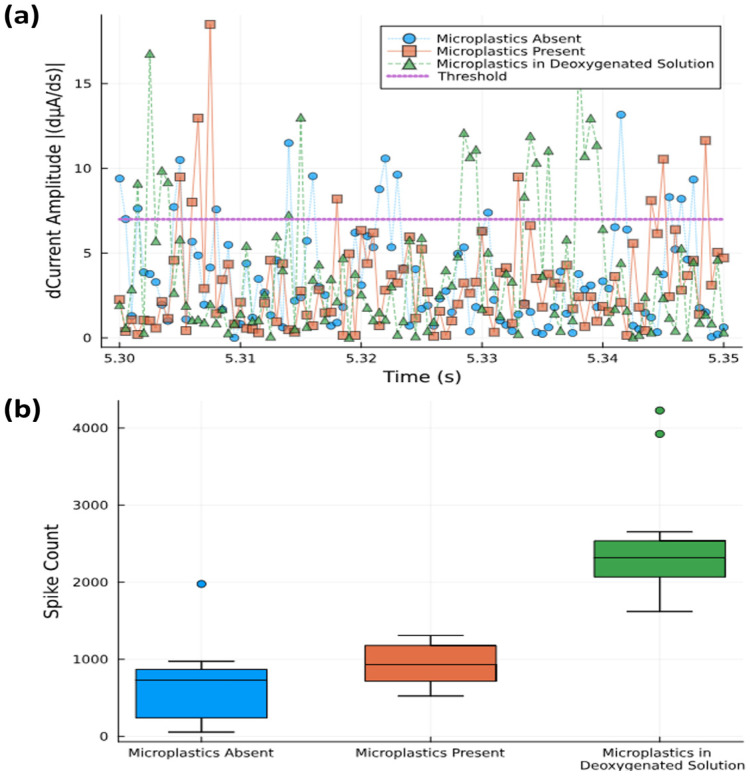
(**a**) Spike counts with threshold level of 7 dµA/ds separating out those that are counted from background noise (0.05 s time window shown). (**b**) Statistical box plot of spike counts above threshold level measured over 10 s time interval.

The FAM signals were recorded with a 40 μm diameter WE (sample 4) and analyzed over a 10 s time frame. The signals were time-differentiated to better distinguish fast variations within the signal concomitant with spikes (Figure 6a). The differentiation of data at 0.05 ms intervals shows spike magnitudes for three different solutions: (i) no microplastics (N = 20), (ii) with 0.02% (*w/v*) microplastics (N = 20), and (iii) a deoxygenated solution with 0.02% (*w/v*) microplastics (N = 20). The samples exceeding a threshold value were counted as spikes and statistically summarized using the Julia programming language.

Box plots were used to show the total spike count within a 10 s time frame for the three different solutions used (Figure 6b). Although the mean values range from 60 counts s^−1^ (no microplastics) to 90 counts s^−1^ (with microplastics), the analysis suggests that spike counting alone does not fully differentiate between solutions with and without microplastics due to the overlapping confidence intervals. The aqueous media’s background oxygen may obscure the changes in the signal brought on by microplastics, and by removing this background through deoxygenation, the spike count was raised to 230 counts s^−1^. This shows that the use of oxygen scavengers to bind dissolved oxygen may not interact with oxygen bound to the microplastic particles themselves (and which is released upon contact with the electrode), thus effectively increasing the signal-to-noise ratio. Thus, although we can quantitatively see a difference between absence and presence, the spike count seems not to be sufficient for quantifying the microplastic concentrations in oxygenated solutions.

### 3.5. FFT Analysis of Resultant Signals

**Figure 7:**
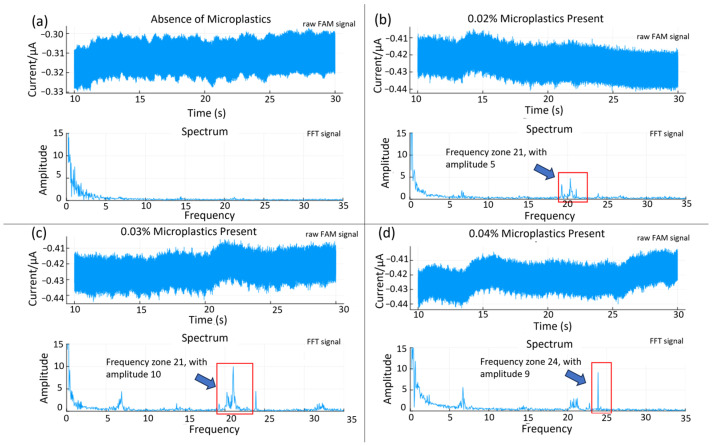
FFT analysis from microplastic concentrations of (**a**) zero, (**b**) 0.02%, (**c**) 0.03%, and (**d**) 0.04% (*w/v*). FFT analysis shows periodic frequency associated with presence of microplastics.

**Figure 8:**
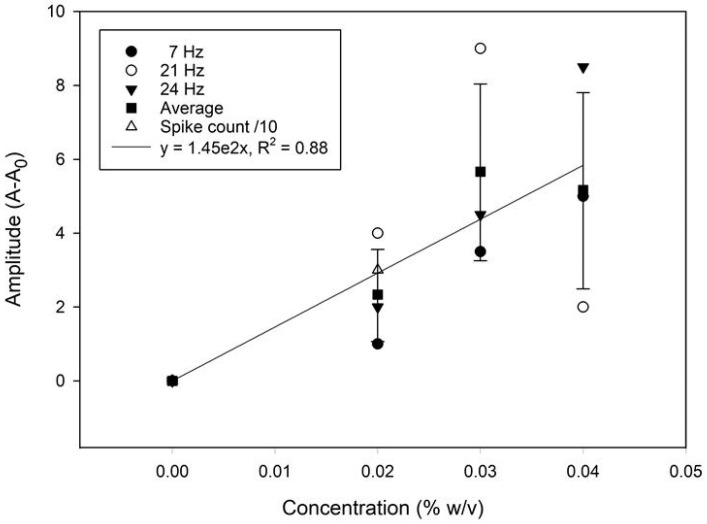
A calibration plot from the signal average (mean) showing the change in the FFT amplitude with the microplastic concentrations. The relative change in the spike counts has been plotted for comparison. The error bars represent the standard error of the mean (±σ, n = 3).

A frequency domain approach was attempted for assessing the quantitative potential of the sensor. The time and frequency domain responses for solutions containing different microplastics loading (0 to 0.04% (*w/v*)) are displayed in Figure 7. Both the amplitude and the frequency of the most prominent peak show a positive correlation with the concentration. A calibration graph based on the average amplitude shift (the relative signal strength derived from the FFT analysis) from the 7, 21, and 24 Hz frequency peaks is presented in Figure 8. The relative change in the spike counts (microplastics present/absent div 10 from Figure 6) has been plotted for comparison. The relative signal increase suggests that the most prominent frequency response increases with the concentration, due to more impacts within the same time frame. This is, however, not expected to be perpetually linear, as an increased concentration will also produce more impacts with adjacent microplastics, resulting in a trajectory shift, loss of momentum, and overall increased sluggishness. Scrutinizing the frequency information, rather than time-domain naïve spike counting, can, therefore, help improve the accuracy in better distinguishing impacts from noise. Moreover, information from both domains could be integrated in a multivariate model for even better performance. This is beyond the scope of the present work.

## 4. Discussion

Employing a standard screen-printed electrode (ZP-HVC-SPE) [46] gave a background signal that was too large compared to the spike events produced by the microplastics, in contrast to a smaller WE of the microwire electrodes. Although the WE presented in this paper had larger diameters than those utilized by Shimizu et al. [45], there were detectable spikes from the microplastic solutions visible in the FAM spike recordings and FFT analysis. Although spikes generated from the 10 µm particles were not visible from direct observations, impacts were visible as “noise” using larger 1 mm PET particles. Hence, the cumulative detection of spikes above a threshold demonstrated by FAM and FFT effectively separated solutions containing microplastics from those without microplastics. This method can, therefore, quantify the overall concentration of microplastic particles, even though it cannot distinguish individual ones.

In the context of early-stage sensor development, where signal variability is expected along with the interference of a significant amount of noise signal, an R^2^ > 0.75 is considered acceptable [50]. Although a higher R^2^ value would indicate better performance, the R^2^ = 0.88 achieved in this study based on the linear regression of the mean is comparable with the results obtained with other low-cost commercial sensors [51]. The phasing in/out of some frequencies (e.g., 21 vs. 24 Hz) with increasing particle concentrations may cause amplitude variations that also reduce the R^2^ term.

The current study did not include a systematic anti-interference validation used to assess the sensor’s selectivity against other particulate matter or chemical species. Only the impact from the background oxygen was investigated by comparison to deoxygenated samples, which supported the hypothesis that microplastics function as oxygen carriers. Thus, it is expected that both chemical and particulate matter with comparable interfering capabilities may be electroactive for the given WE potential. One should, therefore, design a test matrix of components found in real samples (e.g., organic matter, salts, and colloidal particles) in order to identify their impact as part of a future validation study.

Oxygen scavengers such as Na_2_SO_3_ primarily react with dissolved free oxygen and not oxygen adsorbed onto or trapped within the hydrophobic surfaces or internal voids of the microplastic particles. However, the efficacy in removing surface absorbed or entrapped oxygen on microplastics would most likely require more focused investigations. It is possible that the deoxygenation method might have introduced electrochemical effects. The absence of oxygen could alter the redox dynamics in the solution, thus influencing the baseline signals. Additionally, scavenging agents used for deoxygenation might have interacted with the electrode surface, possibly producing some molecular reaction, introducing noise, or affecting reproducibility. Replacing the chemical oxygen scavenger with argon gas for deoxygenation could help reduce potential side reactions caused by the scavenger.

The carbon surface of the WE must remain chemically inert during the oxidative and reductive electrochemical processes so that the anodic and cathodic peaks observed during the carbon electrode’s CV analysis come from the dissolved electrochemical species that are recycled and not from any underlying electrode material. Thus, a characteristic “duck-shaped” plot of the reversible reaction prevails. In contrast, if the underlying electrode material interferes through dissolution or the formation of oxides, non-reversibility exists, causing deviations from the “duck-shaped” plot. This acts as an indication that the carbon coating was compromised, rendering them not suitable for further studies.

Shimizu et al. [45] employed a carbon fiber electrode manufactured using specialized techniques, measuring 1 mm in length and 7 µm in diameter. Since such small-diameter fibers were not readily available, lab wires with a diameter down to 25 µm (40 µm with carbon coating) were chosen for this study. The dynamic range was thus restricted to 100 nA in contrast to the discrete nA level used by Shimizu. Although the individual spikes could not be investigated in the same detail, their presence was analyzed through the means of FAM and FFT.

## 5. Conclusions

Microplastics detection from electrochemical sensors produced from standard lab tools was demonstrated using FAM spike recordings and an FFT analysis. The “impact concept” proved a higher susceptibility for spikes as well as the presence of unique frequency spectra not visible in pure solutions devoid of microplastics. The combination and improvement of both approaches may result in an electrochemical microplastics detection technique that is effective. The electrochemical sensor does not require any specialized knowledge or sophisticated laboratory tools that require excessive space. In contrast to existing detection methods, it provides an affordable, effective, and user-friendly solution that might find broad scientific and commercial adoption by making it portable and submersible in water. By integrating the WE with the lowest possible diameter on a common substrate with a shared CE and RE by screen printing, the protocol would just require a connection to a miniaturized potentiostat tailored to FAM, with FFT analysis software presenting the data in real time.

## Figures and Tables

**Table 1:** Different types of plastics that are commonly found in marine debris [6,18].

Plastic Class	Products and Typical Origin
Low-density polyethylene (LDPE)	Plastic bags, six-pack rings, bottles, netting, straws
High-density polyethylene (HDPE)	Milk and juice jugs
Polypropylene (PP)	Rope, bottle caps, netting
Polystyrene (PS)	Plastic utensils, food containers
Foamed polystyrene	Floats, bait boxes, foam cups
Nylon	Netting and traps
Thermoplastic polyester, i.e., polyethylene terephthalate (PET)	Plastic beverage bottles
Polyvinyl chloride (PVC)	Plastic film, bottles, cups
Cellulose acetate	Cigarette filters
Polypropylene (PP)	Baby milk bottle, food-grade bottle package

**Table 3:** Diameters of fabricated electrodes before and after carbon coating.

Sample No	Conductor	Diameter Pre-Carbon Coating (µm)	Diameter Post-Carbon Coating (µm)
1	Silver	190	210
2	Silver	130	150
3	Copper	50	70
4	Silver	25	40

## Data Availability

The original contributions presented in this study are included in the article. Further inquiries can be directed to the corresponding author(s).
